# Dental Students

**Published:** 1886-11

**Authors:** 


### Editorial, Etc.

---

DENTAL STUDENTS.—The large increase in the number of dental students now in Baltimore is very gratifying to all interested in dental instruction, as this city has been designated the "Mother of the American System of Dental Surgery." When the writer entered the dental profession some thirty years ago, twenty-seven of which have been devoted to dental teaching, the average number of students seeking a professional education was not more than forty, and after waiting for more than a quarter of a century he is at last gratified in being able to announce that at one dental school alone in Baltimore, the *Dental Department of the University of Maryland*, the number of students now present is *one hundred and twenty.*

It is but natural that Baltimore, where the first effort was made to rescue the profession of dentistry from a mere pursuit followed by uneducated men, should become the acknowledged centre of dental education, and that her schools should be patronized by those who care more for what they acquire in the form of knowledge, than in the form and name of being a "Doctor of Dental Surgery."

When the writer first became connected with a dental school, there were but three in existence, now such schools can be found in every part, almost, of this country. Outside of the large cities, the mistake of establishing such schools is apparent in the lack of material for practical experience. To furnish such material, apart from that portion of the inhabitants able and willing to pay the usual fees to local practitioners, requires a large population, and to endeavor to teach dentistry without a large supply of Infirmary patients is to utterly fail in such an undertaking. Small fees may be an inducement, but such fees become more costly than larger ones, if the facilities for practical experience cannot be furnished. With a population approaching nearly to half a million, the supply of clinical material is necessarily large, and Baltimore cannot fail to meet this important and essential demand of her dental schools.

